# The global prevalence of primary central nervous system tumors: a systematic review and meta-analysis

**DOI:** 10.1186/s40001-023-01011-y

**Published:** 2023-01-20

**Authors:** Nader Salari, Hooman Ghasemi, Reza Fatahian, Kamran Mansouri, Sadat Dokaneheifard, Mohammad hossain Shiri, Mahvan Hemmati, Masoud Mohammadi

**Affiliations:** 1 Department of Biostatistics, School of Health, Kermanshah University of Medical Sciences, Kermanshah, Iran; 2 Student Research Committee, Kermanshah University of Medical Sciences, Kermanshah, Iran; 3 Department of Neurosurgery, School of Medicine, Kermanshah University of Medical Sciences, Kermanshah, Iran; 4 Medical Biology Research Centre, Kermanshah University of Medical Sciences, Kermanshah, Iran; 5 Department of Human Genetics, Sylvester Comprehensive Cancer Center, University of Miami Miller School of Medicine, Miami, FL 33136 USA; 6 Cellular and Molecular Research Center, Gerash University of Medical Sciences, Gerash, Iran

**Keywords:** CNS tumors, Prevalence, Brain tumor, Systematic review

## Abstract

**Background:**

Primary central nervous system (CNS) tumors are a heterogeneous group of neoplasms, including benign and malignant tumors. Since there are many heterogeneities in the prevalence reported in previous studies on this type of tumor, this study was performed to determine the overall prevalence of different primary CNS tumors.

**Method:**

The study was conducted as a systematic review and meta-analysis by searching international databases, including PubMed, Scopus, Science Direct, Web of science, and the Google Scholar search engine until August 2020. After transferring the studies to information management software (EndNote) and eliminating duplicate studies, the remaining studies were reviewed based on inclusion and exclusion criteria according to three stages of primary and secondary evaluation and qualitative evaluation. Comprehensive Meta-Analysis software, Begg, Mazumdar, and I^2^ tests were used for data analysis, publication bias analysis, and heterogeneity analysis, respectively.

**Results:**

After performing the systematic review steps, 80 studies were included for final analysis. Based on 8 studies, the prevalence of brain tumors was 70.9%. Also, studies on 7 other studies showed that the prevalence of spinal tumors was 12.2%. A review of 14 studies showed that the prevalence of neuroepithelial tumors was 34.7%. The analysis of 27 studies reported a prevalence of glioma tumors of 42.8%. Analyses performed on other studies showed that the prevalence of pituitary adenomas was 12.2%, embryonal tumors 3.1%, ependymal tumors 3.2%, meningiomas 24.1%, glial tumors 0.8%, astrocytic 20.3%, oligodendroglial 3.9%, glioblastoma 17.7%, schwannoma 6.7%, medulloblastoma 7.7% and Polycystic astrocytomas 3.8%.

**Conclusion:**

As a result, it can be stated that brain tumors are the most common type of primary CNS tumors. It was also observed that tumors involving neuroepithelial cells are more common in patients than other types of tumors.

## Related studies

Studies in the field of primary central nervous system tumors are broad, and so far, no study has collected data from these extensive studies. Certainly, determining the general prevalence of nervous system tumors can make policy measures in the prevention, diagnosis, and treatment of tumors effective and reduce treatment costs.

## Background

Primary tumors of the central nervous system (CNS) are a heterogeneous group of neoplasms that include benign and malignant tumors [1], which are known as tumors in the brain and spinal cord [2]. Various factors such as age, race, ethnicity, gender, environmental factors, hormones, and genetics can play a role in the etiology of CNS tumors [3]. More than 100 types of tumors that are histologically different are known as subtypes of CNS tumors. The incidence of each tumor varies with age and tissue involved. These tumors include glioma, astrocytoma, embryonal tumors, meningioma, and medulloblastoma [4]. Pituitary and pineal gland tumors are other CNS tumors [2]. The most common malignant tumor among CNS tumors is glioblastoma which has the highest mortality rate. On the other hand, meningioma is known as the most common benign tumor [5].

CNS tumors are not as common as other tumors, such as gastrointestinal cancers. However, the number of people with CNS tumors has increased over time [6]. CNS tumors are the most common type of cancer and the second leading cause of death at the age of 19 in the United States and Canada [7].

A 2020 study of 242 Indian children found that boys were more likely to be infected than girls. This study stated that the cerebellum was the most common site of tumors in the studied samples, followed by the brain’s hemispheres with the highest incidence of tumors [8].

Generally, primary malignancies account for about 2% of all cancers. About half of CNS tumors are benign. However, if benign tumors are not operable and radiotherapeutic, they can be fatal due to growth in the closed space of the skull [9]. A population-based study in 2019 stated that 5.5 out of 100.00 people develop glioma [10]. Another study in Iran stated that 6 out of 100,000 people are diagnosed with CNS tumors [11].

CNS tumors are recognized as one of the leading causes of death in children and adults [12]. In a way, these tumors are the second leading cause of death in children and the third leading cause of death in adults [9]. Also, the complications of this disease have a great impact on the individual, family, and social lifestyle of patients [13].

CNS tumors increase the pressure inside the skull or the spinal cord by stimulating or destroying adjacent nerve tissue and spreading the mass in a constant volume, which causes symptoms. A histological type of CNS tumor may show different clinical symptoms depending on the anatomical location involved. Therefore, it seems difficult to diagnose the exact type of tumor and its malignancy based on clinical signs [14].

There are several factors associated with CNS tumors that should be considered, including the choice of a new and appropriate biological treatment method and the effect of the natural history of brain development on the nature of the disease [13]. CNS tumors can cause mental alteration and neurological disorders and put a heavy burden on families and the health system [2].

Because CNS tumors are very diverse and cause different complications in different people and cause severe disabilities in a person, early diagnosis of tumor type is very important. Also, since there is a lot of heterogeneity due to previous studies on this type of tumor, the present study is conducted to investigate the prevalence of different types of primary CNS tumors.

## Method

### Protocol and information resources

This study was performed by systematic review method and following PRISMA (Preferred Reporting Items for Systematic Review and Meta-Analysis) [15], and it examines the prevalence of primary CNS tumors worldwide. The search was conducted in the following databases up to 29 August 2020: PubMed, Scopus, Science Direct, and Web of science (WoS).

### Search strategy

Initially, a comprehensive study was conducted to select appropriate terms around the title. After selecting keywords appropriate to this systematic review study, a search was conducted in the databases. To access the articles, the keywords; central nervous system tumors, primary brain tumors, spinal cord neoplasm, glioma, meningioma, glioblastoma, oligodendroglioma, medulloblastoma, astrocytoma, prevalence, cross-sectional, and outbreak were used. The articles were collected in Endnote software after performing the search without any time limit. To maximize the search comprehensiveness, the list of sources used in all related articles found in mentioned search was manually reviewed.

### Inclusion and exclusion criteria of the study

The inclusion and exclusion criteria of this study were designed based on PICOS guidelines. Inclusion criteria for this study included: 1- studies that examined the prevalence of primary CNS tumors, 2- observational studies, and 3- cross-sectional studies. Exclusion criteria included: 1- unrelated study, 2- studies without sufficient data, 3- duplication studies, 4- unclear study methods, 5- interventional studies, 6- case report studies, and 7- studies for which the full text is not available.

### Selection and extraction of studies

After transferring all the extracted studies to EndNote software, duplicate articles that were identified were removed. the researchers reviewed the studies by title and abstract by the defined inclusion and exclusion criteria. during the second evaluation process, the full text of the remaining articles was re-examined based on the inclusion and exclusion criteria. During these steps, studies were performed by two researchers independently to minimize bias. If there was a disagreement between the two researchers, the studies were conducted by a third party. After these steps, the approved studies entered the qualitative evaluation stage to evaluate the methodological quality. Information on all final articles submitted to the systematic review and meta-analysis process is extracted from a pre-prepared checklist. This checklist the included article title, first author name, year of publication, place of study, sample size, sample evaluation method, gender, type of study, study population, number of people with CNS tumors in general, and the tumor type.

### Qualitative evaluation of studies

Since cross-sectional observational studies were considered the inclusion criteria, the STROBE checklist was used to critique and evaluate the quality of articles approved in the previous stages. This checklist has 22 items, some of which have several sections, so the STROBE checklist contains a total of 32 items that examine different parts of the study body, including the title, abstract, introduction, data collection methods, statistical analysis methods, and presentation of results. Articles that have lost more than 50% of the items defined in the STROBE checklist are considered as poor quality articles due to their high probability of bias and were excluded from the study, so at this stage, studies that were considered qualitatively as studies of good and average methodological quality entered the analysis process. In the present study, based on the evaluation made based on the STROBE checklist, 78 articles were entered into the systematic review and meta-analysis process as good and medium methodological quality studies.

### Statistical analysis

The I^2^ test was used to evaluate the heterogeneity of the selected studies. To investigate the dissemination error due to the high volume of samples included in the study, the Egger test was applied at a significant level of 0.05 and the corresponding funnel plot. Data analysis was performed using Comprehensive Meta-Analysis software (Version 2).

## Results

**Fig. 1:**
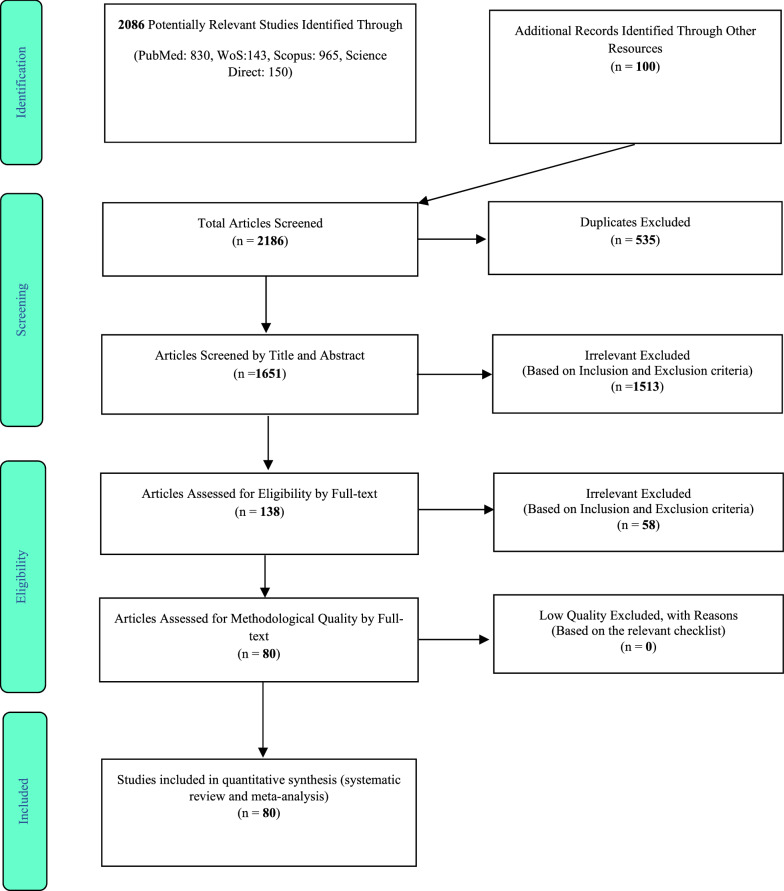
Flowchart of PRISMA

In this systematic review and meta-analysis study, the information of studies conducted about the prevalence of primary central nervous system (CNS) tumors in the world until 29 August 2020 was systematically reviewed according to PRISMA guidelines. Based on the initial search in the database, 2186 possible related articles were identified and transferred to the information management software (EndNote). 535 out of 2186 identified studies were duplicated and excluded. In the screening phase, out of 1651 studies, the remaining 1513 articles were removed by studying the titles and abstracts based on inclusion and exclusion criteria. In the competency evaluation stage, out of 138 studies, the remaining 60 articles were removed by studying the full text of the article based on inclusion and exclusion criteria due to irrelevance. In the qualitative evaluation stage, all 80 studies were confirmed by reading the full text of the article and based on the score obtained from the STROBE checklist (Fig. 1; Table 1).

**Table 1:** Information of articles

Rows	Name [references]	Country	Year of publication	Participants	Prevalence
1	Seung Hoon Lee [34]	South Korea	2017	654	Germ cell tumors (pituitary adenomas: 6.7%, pineal tumor: 34.5%, Brain tumor: 35%)
2	Yeon-Lim Suh [25]	Korea	2002	3221	Pituitary adenomas: 19%, meningioma: 24%, tumors of neuroepithelial tissue: 31.6%(embryonal tumors: 2.6% MEDULLOBLASTOMA: 1.8%, ependymal tumors: 1.8%, glial tumor of CNS and pineal gland: 2.3%, astrocytic tumors of CNS: 8.9%, oligodendroglial: 3.3%, choroid plexus: 0.4%, glioblastoma: 10.6%), germ cell tumors: 1.7%
3	Yeon-Lim Suh [25]	Korea	2002	2556	Pituitary adenomas: 15.1%, meningioma: 25.1%, tumors of neuroepithelial tissue: 34.9%(glioblastoma: 4.8%, glial tumor of CNS and pineal gland: 0.35%, astrocytic tumors of CNS: 15.9%, oligodendroglial: 4.6%, choroid plexus: 0.4%, embryonal tumors: 3.3%, ependymal tumors: 2.4%) germ cell tumors: 1.7%
4	Anirban Ghosh [42]	India	2004	873	Brain tumor [glioma: 60% (ependymal tumors: 3.2%, astrocytic tumors of CNS: 36.7%, oligodendroglial: 4.9%), meningioma: 11.6%, pineal tumor: 0.2%, medulloblastoma: 1.6%, pituitary adenomas: 4.1%]
5	Axel Tjo¨ rnstrand [43]	Sweden	2014	592	Pituitary adenomas: 100%
6	Adrian F. Daly [44]	Belgium	2006	68	Pituitary adenomas: 100%
7	Kullapat Veerasarn [45]	Thailand	2016	93,810	Pituitary adenomas: 3.8%, meningioma: 15.3%, pineal tumor: 1.9%
8	Kurupath Radhakrishnan [46]	USA	1995	339	Pituitary adenomas: 16.2%, glioma: 29.4% (ependymal tumors: 1.1%, astrocytic tumors of CNS: 24.7%, oligodendroglial: 3.2%), meningioma: 40.1%, schwannoma: 4.4%, medulloblastoma: 1.7%
9	T.B. Johannesen [36]	Norway	2004	14,641	Pituitary adenomas: 9.3%, meningioma: 21.9%, pineal tumor: 0.7%, brain tumor: 60.1%, spinal tumor: 1.9%
10	Yun-Sik Dho [41]	Republic of Korea	2017	11,827	10 pituitary adenomas: 17.9%, meningioma: 37.2%, tumors of neuroepithelial tissue: 13.6% (glioma: 12.6%, ependymal tumors: 0.9%, glial tumor of CNS and pineal gland: 1.07%, astrocytic tumors of CNS: 2.03%, oligodendroglial: 1.2%, choroid plexus: 0.16, glioblastoma: 5.3%, pilocytic astrocytomas : 0.4%), germ cell tumors: 1.07%
11	Ahmad F. Tamimi [47]	Jordan	2015	313	Pituitary adenomas: 9.2%, embryonal tumors: 7.02 (medulloblastoma: 4.7%), meningioma: 26.1%, tumors of neuroepithelial tissue: 37.6 (ependymal tumors: 1.5%, astrocytic tumors of CNS: 9.2%, oligodendroglial: 1.2%, choroid plexus: 0.3%, glioblastoma: 18.8%, pilocytic astrocytomas: 3.8%), schwannoma: 3.8%
12	Leonard T. Kurland [48]	USA	1982	223	Pituitary adenomas: 13%, glioma: 34.9%, meningioma: 39.4%
13	Leonard T. Kurland [48]	USA	1982	189	Pituitary adenomas: 12.1%, glioma: 35.9%, meningioma: 39.6%
14	Chang-Hyun Lee [2]	Korea	2010	5,692	Pituitary adenomas: 13.8, meningioma: 29.6%, tumors of neuroepithelial tissue: 19.3% (glioma: 1.9%, embryonal/primitive /medulloblastoma: 1.2%, ependymal tumors: 1.1%, glioblastoma: 5.9%, astrocytic tumors of CNS: 4.8%, oligodendroglial: 1.05%, choroid plexus: 0.26%)
15	Haley Gittleman [16]	USA	2014	51,125	Pituitary adenomas: 100%
16	NUNG WON CHOI [49]	USA	1970	760	Pituitary adenomas: 2.6%, glioma: 56.7% (ependymal tumors: 2.8%, astrocytic tumors of CNS: 17.5%, Oligodendroglial: 1.3%, glioblastoma: 20.9%, medulloblastoma: 2.5%), meningioma: 6.8%
17	David Gigineishvili [21]	Georgia	2013	980	Pituitary adenomas: 11.7%, meningioma: 25.9%, tumors of neuroepithelial tissue: 13.1% (glioblastoma: 4.8%, ependymal tumors: 0.4%, astrocytic tumors of CNS: 3.3%, oligodendroglial: 1.22%)
18	D.Gigineishvili [50]	Georgia	2013	473	Pituitary adenomas: 17.9%, meningioma: 45.2%, glioblastoma: 9.9%
19	Ramandeep S. Arora [51]	England	2009	54,336	Pituitary adenomas: 8.8%, meningioma: 15.8%, pineal tumor: 0.38%, tumors of neuroepithelial tissue: 53% (embryonal tumors: 1.5% (medulloblastoma: 1.07%), ependymal tumors: 1.96%, astrocytic tumors of CNS: 35.2% (glioblastoma: 21.7%, pilocytic astrocytomas: 1.68%), oligodendroglial: 12.2%, choroid plexus: 0.18%), tumors of cranial and spinal nerves: 6.8%
20	Alberto Fernandez [52]	England	2010	63	Pituitary adenomas: 100%
21	Giuseppe D 'Alessandro [53]	Italy	1995	178	Pituitary adenomas: 14%, meningioma: 37%, tumors of neuroepithelial tissue: 34.8%
22	Amélie Darlix [54]	France	2017	57,816	Pituitary adenomas: 3.7%, meningioma: 7.1%, tumors of neuroepithelial tissue: 43.5% (glioma: 39.1%, embryonal tumors: 1.6% (medulloblastoma: 0.95%), ependymal tumors: 2.3%, astrocytic tumors of CNS: 26.5% (glioblastoma: 21.4%), oligodendroglial: 5.9%, choroid plexus: 0.3%), germ cell tumors: 0.4%, schwannoma: 8.6%
23	Hideo Nakamura [39]	Japan	2011	5,448	Pituitary adenomas: 17.8%, glioma: 19.5%, meningioma: 36.7%, schwannoma: 9.9%, medulloblastoma: 0.45%
24	Dale L. Preston [37]	Japan	2002	467	Pituitary adenomas: 7.49%, glioma: 9.2%, meningioma: 18.8%, schwannoma: 11.3%
25	Charles A. Stiller [17]	Great Britain (England, Scotland and Wales)	2019	4166	Pituitary adenomas: 1.6%, embryonal tumors: 2.6%, ependymal tumors: 6.98%, meningioma: 1.2%, pineal tumor: 1.1%, astrocytic tumors of CNS: 40.9% (glioblastoma: 3.5%, pilocytic astrocytomas: 20.6%) oligodendroglial: 0.8%, choroid plexus: 2.6%, germ cell tumors: 4%, atypical teratoid/rhabdoid tumors: 1.94%, medulloblastoma: 12.5%
26	Tanya S. Surawicz [55]	USA	1999	20,765	Pituitary adenomas: 8%, meningioma: 25.3%, tumors of neuroepithelial tissue: 51.2% (glioma: 3.6%, embryonal tumors: 1.8%, ependymal tumors: 2.36%, glial tumor of CNS and pineal gland: 1.2%, astrocytic tumors of CNS: 8.1%, oligodendroglial: 2.6%, choroid plexus: 0.3%, glioblastoma: 22.6%, medulloblastoma: 1.8%, pilocytic astrocytomas: 1.78%), spinal tumor and tumors of cranial nerves: 6.5%
27	Emily A.J. Sehmer [19]	England	2014	435	Glioma (astrocytic tumors of CNS: 9.6% (glioblastoma: 86.4%), oligodendroglial: 0.4%)
28	Luc Bauchet [20]	France	2009	1017	Glioma: 51.7% (astrocytic tumors of CNS: 32.3% (glioblastoma: 2.3%, pilocytic astrocytomas: 23.1%), oligodendroglial: 6.5%, ependymal tumors: 8.4%), embryonal tumors: 19% (medulloblastoma: 14.9, atypical teratoid/rhabdoid tumors: 1.08%), glioneuronal tumor: 8.3%, meningioma: 2.3%, pineal gland: 1.08%, choroid plexus: 2.8%, germ cell tumors: 3.5%, Schwannoma: 2.3
29	David J. Cote [56]	USA	2019	97,810	Glioma (ependymal tumors: 6.7%, nonglioblastoma astrocytomas: 20.4, oligodendroglial: 8%, glioblastoma: 57.4%)
30	S Preston-Martin [57]	USA	1989	8612	Glioma: 50.1% (ependymal tumors: 1.3%, astrocytic tumors of CNS: 23.1%, oligodendroglial: 1.5%, glioblastoma: 18.2%, medulloblastoma: 2.2%), meningioma: 28%
31	K. Gousias [58]	Greece	2009	56	Glioma: 100%
32	Faith G. Davis [32]	USA	2001	6908	Glioma: 3.5%, embryonal tumors: 1.4%, ependymal tumors: 1.3%, meningioma: 26.5%, astrocytic tumors of CNS: 6.3%, oligodendroglial: 1.7%, glioblastoma: 22.3%, pilocytic astrocytomas: 1.3%
33	Yoshikazu Okamoto [59]	Switzerland	2004	122	Glioma (oligodendroglial: 40.9%)
34	Emmanuel Desandes [33]	France	2014	3886	Glioma: 6.7%, ependymal tumors: 6.4%, meningioma: 1.5%, oligodendroglial: 4.1%, choroid plexus: 2.7%, glioblastoma: 1.6%, germ cell tumors: 5.6%, atypical teratoid/rhabdoid tumors: 2.4%, medulloblastoma: 13.7%, pilocytic astrocytomas: 21.8%
35	Kenneth R. Hess [60]	USA	2004	22,427	Glioma: 2.8%, astrocytic tumors of CNS: 36.6%, oligodendroglial: 6.04%, glioblastoma: 52.02
36	M. P. W. A. Houben [61]	the Netherlands	2006	11,812	Glioma: (ependymal tumors: 3.2%, oligodendroglial/mixed glioma: 11.8%)
37	Helle Collatz Christensen [18]	DENMARK	2003	11,935	Glioma: 100% (meningioma: 40.59)
38	Janhvi Jaiswal [27]	India	2016	4295	Glioma: 34.1%, ependymal tumors: 2.2%, astrocytic tumors of CNS: 3.1%, oligodendroglial: 9.5%, glioblastoma: 12.9%, pilocytic astrocytomas: 2.07%
39	Faith G. Davis [62]	USA	1996	8,070	Glioma: 5.3%, ependymal tumors: 1.6%, meningioma: 21.1%, pineal tumor: 0.16%, astrocytic tumors of CNS: 19.7%, oligodendroglial: 2.1%, glioblastoma: 25.4%, medulloblastoma: 1.8%, pilocytic astrocytomas: 1.5%
40	Tola MR [63]	Italy	1994	169	Glioma (ependymal tumors: 1.7%, astrocytic tumors of cns: 16.5%, oligodendroglial: 7.6%, glioblastoma: 48.5%)
41	Camille Pouchieu [29]	France	2018	3515	Meningioma: 37.5%, tumors of neuroepithelial tissue: 42.5% (glioma: 4.03%, ependymal tumors: 1.9%, pineal tumor: 0.08%, astrocytic tumors of CNS: 4.2%, oligodendroglial: 0.79%, choroid plexus: 0.28%, glioblastoma: 26.8%, pilocytic astrocytomas: 0.96%), germ cell tumors and cysts: 0.5%, cranial and spinal nerve tumors: 12%,
42	Kimberly R. Porter [64]	USA	2010	18 037	Glioma: 33.2%, meningioma: 32.8%
43	Susan preston-martin [65]	USA	1990	462	Glioma: 28.3%, ependymal tumors: 15.1%, meningioma: 42.8%, astrocytic tumors of CNS: 11.25%
44	Adele Caldarella [30]	Italy	2011	4,417	Embryonal tumors: 0.7%, ependymal tumors: 0.9%, meningioma: 27.8%, pineal tumor: 0.02%, astrocytic tumors of CNS: 25.3%, oligodendroglial: 1.3%, choroid plexus: 0.02%, germ cell tumors: 0.02%
45	Rafael Fuentes-Raspall [66]	Spain	2011	679	Embryonal tumors: 3.5%, ependymal tumors: 1.9%, Astrocytic tumors of CNS: 35.7%, oligodendroglial tumors and mixed histologies: 2.5%, choroid plexus: 0.14%
46	Sarah Khan [67]	United Arab Emirates	2020	744	Embryonal tumors: 13.03%, ependymal tumors: 6.18%, meningioma: 4.1%, astrocytic tumors of CNS: 69.6%(diffuse astrocytic and oligodendroglial tumors: 64.9%), choroid plexus: 0.5%, germ cell tumors: 1.7%
47	Emanuele Crocetti [26]	Austria, Iceland, Ireland, Malta, Norway, Slovakia, Slovenia, Sweden, Northern Ireland, Scotland and Wales and Other 10 countries	2012	44,947	Embryonal tumors: 4.1%, ependymal tumors: 3.59%, astrocytic tumors of CNS: 85.9%, oligodendroglial: 6.4%, choroid plexus: 0.1%
48	Gillian C. Cole [68]	Wales	1989	526	Ependymal tumors: 4.7%, pineal tumor: 0.9%, astrocytic tumors of CNS: 52.6%, oligodendroglial: 6.8%, germ cell tumors: 0.7%, medulloblastoma: 1.7%
49	Kate A. Schellinger [22]	USA	2008	3,226	Spinal tumor: 69.9% (ependymal tumors: 23%, meningioma: 29%)
50	Linh M. Duong [69]	USA	2012	11,712	Ependymal tumors: 21.1%, meningioma: 32.6%, spinal tumor: 26.6%, pilocytic astrocytomas: 1.6%
51	Naseem Ahmed [70]	Pakistan	2007	81	Ependymal tumors: 9.8%, astrocytic tumors of CNS: 35.8%, oligodendroglial: 1.2%, glioblastoma multiforme: 3.7%, pilocytic astrocytomas: 14.8%
52	Peter Kaatsch [24]	Germany	2001	3268	Ependymal tumors: 10.3%, meningioma: 1.16%, pineal tumor: 1.3%, astrocytic tumors of CNS: 41.7%
53	Therese A. Dolecek [23]	USA	2015	51,065	Meningioma: 100%
54	Bernd Holleczek [71]	Germany	2019	992	Meningioma: 100%
55	S. Zouaoui [72]	France	2018	13,038	Meningioma: 100%
56	Luis Eduardo Werneck de Carvalho [73]	Brazil	2017	949	Meningioma: 24.9%, tumors of neuroepithelial tissue: 40%, germ cell tumors: 0.9%
57	Mousa Taghipour [74]	Iran	2010	371	Meningioma: 100%
58	Lars Klaeboe [75]	Denmark, Finland, Norway and Sweden	2005	18,630	Meningioma: 100%
59	Lona C [31]	Italy	1988	182	Meningioma: 28.5%, tumors of neuroepithelial tissue: 56.04%, germ cell tumors: 1.09%
60	CHRISTOPH BURKHARD [40]	Switzerland	2003	196	Pilocytic astrocytomas: 28%
61	Keishi Makino [76]	Japan	2013	6,615	Pineal tumor: 0.46%, germ cell tumors: 1.05%
62	Donna L. Johnston [77]	Canada	2014	574	Medulloblastoma: 100%
63	Yousef S. Khader [35]	Jordan	2018	2096	Brain tumor: 100%
64	Maria Teresa GIORDANA [78]	Italy	1999	45	Medulloblastoma: 68.8%
65	JIANG Tao [79]	China	2011	636	Brain tumor: 100%
66	S. Cordera [80]	Italy	2002	253	Tumors of neuroepithelial tissue: 35.1%
67	Adalberto Miranda-Filho [81]	Brazil, France	2017	78,034	Primary CNS tumors: 100%
68	Sandrine Elia-Pasquet [82]	France	2004	329	Brain tumor: 92.4%, spinal tumor: 7.5%
69	Birthe Krogh Rasmussen [83]	Denmark	2017	1930	Astrocytic tumors of CNS: 15.5%, oligodendroglial: 5.9%, glioblastoma: 70.6%, pilocytic astrocytomas: 1.8%
70	Hyeon Jin Park [84]	Korea	2016	2,116	Germ cell tumors: 54.3%
71	Rose Lai [85]	USA	2008	454	Medulloblastoma: 100%
72	Agne` s Fleury [86]	France	1997	1376	Astrocytic tumors of CNS: 68.3%
73	Abbas Rezaianzadeh [87]	Iran	2020	1043	Brain tumor: 100%
74	Malene Schjønning Nielsen [28]	Denmark	2009	1,304	Oligodendroglial: 100%
75	Marios K. Georgakis [88]	12 SEE countries (Belarus, Croatia, Cyprus, Malta, Montenegro, Greater Poland, Portugal Central, Portugal North, Romania-Cluj, Romania-Iasi, Serbia Central, Slovenia, Turkey-Izmir and Ukraine)	2017	11,438	Primary CNS tumors: 100%
76	Nicolas R. Smoll [38]	Australia	2012	1372	Medulloblastoma: 100%
77	ARE HELSETH [89]	Norway	1995	10,936	Primary CNS tumors: 100%
78	Stefan L¨ONN [90]	Denmark, Finland, Norway, and Sweden	2004	43,120	Primary CNS tumors: 100%
79	Adah S. Zhang [91]	USA	2017	294,666	Brain tumor: 49.9% (glioblastoma: 44.3%, pilocytic astrocytomas: 3.7%)
80	C A STILLER [92]	UK	1994	12,509	Primary CNS tumors: 100%

### Pituitary adenomas

26 studies with a sample size of 331,575 people working on pituitary adenomas were obtained, which its highest prevalence reported by Gittleman et al. [16]. In contrast, the lowest prevalence was reported by Stiller et al. [17] (Table 1). Based on the analysis (I^2^: 99.6) and publication results bias of Begg and Mazumdar rank correlation test analysis at a significance level of 0.1, heterogeneity of the study was not significant (p: 0.566) Meta-analysis, according to which the prevalence of pituitary adenomas was 12.2 (95% CI 9.4–15.7) (Table 2).

**Table 2:** Heterogeneity, publication bias, and the overall prevalence of tumors studied in the study based on meta-analysis and random analysis

Tumor type	Number of articles	Sample size	Heterogenicity (I^2^)	Publication bias (Begg and Mazumdar rank correlation test)	Prevalence (95% CI)
Pituitary adenomas	26	331,575	99.6	0.566	12.2 (95% CI: 9.4–15.7)
Glioma	27	303,967	99.9	0.113	42.8 (95% CI: 29–57.7)
Embryonal tumors	14	207,577	99.3	0.742	3.1 (95% CI: 2.1–4.5)
Ependymal tumors	33	379,800	99.7	0.258	3.2 (95% CI: 2.3–4.4)
Meningioma	42	450,109	99.7	0.438	24.1 (95% CI: 20.5–28.1)
Glial tumor of CNS and pineal gland	17	228,500	99.4	0.692	0.8 (95% CI: 0.4–1.5)
Astrocytic tumors of CNS	33	375,302	99.9	0.744	20.3 (95% CI: 15–26.8)
Oligodendroglial	34	387,350	99.5	0.802	3.9 (95% CI: 3.1–4.9)
Choroid plexus	16	219,897	98.5	0.444	0.4 (95% CI: 0.2–0.7)
Tumors of neuroepithelial tissue	14	162,538	99.8	0.324	34.7 (95% CI: 28.6–41.3)
Glioblastoma	26	616,726	99.9	0.133	17.7 (95% CI: 13.9–22.3)
Germ cell tumors	16	104,207	99.7	0.115	2.6 (95% CI: 0.8–8.2)
Brain tumor	8	314,938	99.2	0.710	70.9 (95% CI: 63.1–77.5)
Spinal tumor	7	108,524	99.9	1.000	12.2 (95% CI: 5–27.1)
Schwannoma	6	65,400	93.3	0.259	6.7 (95% CI: 5.3–8.4)
Medulloblastoma	19	172,593	99.6	0.093	7.7 (95% CI: 4.2–13.6)
Pilocytic astrocytomas	16	427,683	99.7	0.162	3.8 (95% CI: 2.3–6.5)

### Glioma

In the study of glioma tumors, 27 studies with a sample size of 303,967 people were obtained. The highest prevalence of which was reported by Christensen et al. [18], and the lowest prevalence by Semher et al. [19] (Table 1). The heterogeneity of the study was not significant according to the analysis (I^2^ 99.9) and publication bias results based on Begg and Mazumdar rank correlation test analysis at a significance level of 0.1 (p: 0.113). Based on high heterogeneity in the studies, a random-effects model was used in the meta-analysis, according to the prevalence of glioma tumor, which was 42.8 (95% CI: 29–57.7) (Table 2).

### Embryonal tumors

In the study of embryonal tumors, 14 studies with a sample size of 207,577 people were obtained, the highest prevalence of which was reported by Bauchet et al. [20]; however, the lowest prevalence was reported by Gigineishvili et al. [21] (Table 1). Based on analysis (I^2^: 99.3) and the results of diffusion bias according to the Begg and Mazumdar rank correlation test analysis at a significance level of 0.1, the heterogeneity of the study was not significant (p: 0.742). Based on high heterogeneity in the studies, a random-effects model was used in the meta-analysis, according to which the prevalence of embryonal tumors was 3.1 (95% CI: 2.1–4.5) (Table 2).

### Ependymal tumors

33 studies with a sample size of 379,800 people were obtained working on ependymal tumors, showing the highest prevalence of which was reported by Schlinger et al. [22] and the lowest prevalence by Jiginishvili et al. [21] (Table 1). A study of the heterogeneity of these researches showed the result was insignificant (p: 0.258) based on analysis (I^2^: 99.7) and diffusion bias results based on Begg and Mazumdar rank correlation test analysis at a significance level of 0.1. Based on high heterogeneity in the studies, a random-effects model was used in the meta-analysis, according to which the prevalence of ependymal tumors was 3.2 (95% CI: 2.3–4.4) (Table 2).

### Meningioma

In the study of meningioma tumors, 42 studies with a sample size of 450,109 were obtained. The highest prevalence of which was reported by Dolesk et al., [23], and the lowest prevalence by Katesh et al. [24] (Table 1). The heterogeneity study of the reports was insignificant (p: 0.438) according to the analysis (I^2^: 99.7) and diffusion bias results based on Begg and Mazumdar rank correlation test analysis at a significance level of 0.1. Based on high heterogeneity in the studies, a random-effects model was used in the meta-analysis, according to which the prevalence of meningioma was 24.1 (95% CI: 20.5–28.1) (Table 2).

### Glial tumor of CNS and pineal gland

In the study of glial tumors of CNS and pineal gland, 17 studies with a sample size of 228,500 people were obtained, showing the highest prevalence of which was reported by Lim Soo et al. [25], and the lowest prevalence was reported by Lim Soo et al. [25] (Table 1). Investigation of heterogeneity in the studies was not significant (p: 0.692), based on analysis (I^2^: 99.4) and publication bias results based on Begg and Mazumdar rank correlation test analysis at a significance level of 0.1. Based on high heterogeneity in the studies, a random-effects model was used in the meta-analysis, according to which the prevalence of astrocytic tumors of the CNS was 0.8 (95% CI: 0.4–1.5) (Table 2).

### Astrocytic tumors of CNS

In the study of astrocytic tumors of the CNS, 33 studies were obtained with a sample size of 375,302 people, demonstrating the highest prevalence of which was reported by Croust et al. [26]. In contrast, the lowest prevalence was reported by Jesual et al. [27] (Table 1). A study of the heterogeneity of the research showed the result was not significant (p: 0.744), based on analysis (I^2^: 99.9) and publication bias results based on Begg and Mazumdar rank correlation test analysis at a significance level of 0.1. Based on high heterogeneity in studies of the random-effects model, Meta-analysis was used, according to which the prevalence of astrocytic tumors of the CNS was 20.3 (95% CI: 15–26.8) (Table 2).

### Oligodendroglial

In the study of the oligodendroglial tumor, 34 studies with a sample size of 387,350 people were obtained, the highest prevalence of which was reported in the study of Nielsen et al. [28] and the lowest prevalence in the study of Poschio et al. [29] (Table 1). Based on analysis (*I*^2^: 99.5) and publication bias results according to Begg and Mazumdar rank correlation test analysis at a significance level of 0.1, it was shown that heterogeneity of these studies was not significant (*p*: 0.802). Based on high heterogeneity in the studies, a random-effects model was used in the meta-analysis, according to which the prevalence of oligodendroglial tumor was 3.9 (95% CI: 3.1–4.9) (Table 2).

### Choroid plexus

In the study of choroid plexus tumors, 16 studies with a sample size of 219,897 people were obtained, the highest prevalence of which was reported by Basht et al. [20], and the lowest prevalence was reported by Caldarella et al. [30] (Table 1). Study heterogeneity of the reports revealed no significant result (p: 0.444) based on analysis (*I*^2^: 98.5) and publication bias results based on Begg and Mazumdar rank correlation test analysis at a significance level of 0.1. Based on high heterogeneity in the studies, a random-effects model was used in the meta-analysis, according to which the prevalence of choroid plexus tumor was 0.4 (95% CI: 0.2–0.7) (Table 2).

### Tumors of neuroepithelial tissue

In the study of tumors of neuroepithelial tissue, 14 studies with a sample size of 162,538 people were obtained, the highest prevalence of which was reported by Luna et al. [31], and the lowest prevalence was reported by Jiginishvili et al. [21] (Table 1). Based on analysis (*I*^2^: 99.8) and publication bias results obtained by Begg and Mazumdar rank correlation test analysis at a significance level of 0.1, investigation of the heterogeneity showed no significant data (*p*: 0.324). Based on high heterogeneity in the studies, a random-effects model was used in the meta-analysis, according to which the prevalence of tumors of neuroepithelial tissue was 34.7 (95% CI: 28.6–41.3) (Table 2).

### Glioblastoma

In the study of glioblastoma tumors, 26 studies with a sample size of 616,726 people were obtained, showing the highest prevalence of which was reported by Davis et al. [32] and the lowest prevalence was reported by Desandes et al. [33] (Table 1). Study heterogeneity based on analysis (*I*^2^: 99.9) and publication bias results based on Begg and Mazumdar rank correlation test analysis at a significance level of 0.1 showed no significant result (*p*: 0.133). Based on high heterogeneity in the studies, a random-effects model was used in the meta-analysis, according to which the prevalence of glioblastoma tumor was 17.7 (95% CI: 13.9–22.3) (Table 2).

### Germ cell tumors

In the study of germ cell tumors, 16 studies were obtained with a sample size of 104,207 people, revealing the highest prevalence of which was reported by Lee et al. [34] and the lowest prevalence by Calderla et al. [30] (Table 1). The heterogeneity of the study was not significant (*p*: 0.115) based on analysis (*I*^2^: 99.7) and results of publication bias based on the analysis of the Begg and Mazumdar rank correlation test at a significance level of 0.1. Based on high heterogeneity in the studies, a random-effects model was used in the meta-analysis, according to which the prevalence of germ cell tumors was 2.6 (95% CI: 0.8–8.2) (Table 2).

### Brain tumor

In the study done on a brain tumor, 8 studies with a sample size of 314,938 people were obtained, revealing the highest prevalence of which was reported by Ghader et al. [35] and the lowest prevalence was reported by Lee et al. [34] (Table 1). Study heterogeneity based on analysis (*I*^2^: 99.2) and publication bias results based on Begg and Mazumdar rank correlation test analysis at a significance level of 0.1 showed no significance (*p*: 0.710). Based on high heterogeneity in the studies, a random-effects model was used in the meta-analysis, according to which the prevalence of brain tumors was 70.9 (95% CI: 63.1–77.5) (Table 2).

### Spinal tumor

In the study performed on the spinal tumor, 7 studies with a sample size of 108,524 people were obtained, the highest prevalence of which was reported by Schillinger et al. [22], and the lowest prevalence was reported by Johansen et al. [36] (Table 1). Study heterogeneity based on analysis (*I*^2^: 99.9) and publication bias results based on Begg and Mazumdar rank correlation test analysis at a significance level of 0.1 showed no significance (*p*: 1.000). Based on high heterogeneity in the studies, a random-effects model was used in the meta-analysis, according to which the prevalence of spinal tumor was 12.2 (95% CI: 5–27.1) (Table 2).

### Schwannoma

In the study of schwannoma tumor, 6 studies with a sample size of 65,400 people were obtained, showing the highest prevalence of which was reported by Preston et al. [37] and the lowest prevalence by Basht et al. [20] (Table 1). Study heterogeneity based on analysis (*I*^2^: 93.3) and publication bias results based on Begg and Mazumdar rank correlation test analysis at a significance level of 0.1 showed no significance (*p*: 0.259). Based on high heterogeneity in the studies, a random-effects model was used in the meta-analysis to determine the prevalence of schwannoma tumor was 6.7 (95% CI: 5.3–8.4) (Table 2).

### Medulloblastoma

In the study performed on medulloblastoma tumor, 19 studies with a sample size of 172,593 people were achieved, showing the highest prevalence of which was reported by Small et al. [38] and the lowest prevalence was reported by Nakamura et al. [39] (Table 1). Study heterogeneity based on analysis (*I*^2^: 99.6) and publication bias results based on Begg and Mazumdar rank correlation test analysis at a significance level of 0.1 showed no significance (*p*: 0.093). Based on high heterogeneity in the studies, a random-effects model was used in the meta-analysis, according to which the prevalence of medulloblastoma was 7.7 (95% CI: 4.2–13.6) (Table 2).

### Pilocytic astrocytomas

In the study done on pilocytic astrocytomas, 16 studies with a sample size of 427,683 people were obtained, clarifying the highest prevalence of which was reported by Burkard et al. [40] and the lowest prevalence was reported by Doo et al. [41] (Table 1). Study heterogeneity based on analysis (*I*^2^: 99.7) and publication bias results based on Begg and Mazumdar rank correlation test analysis at a significance level of 0.1 showed no significance (*p*: 0.162). Based on high heterogeneity in the studies, a random-effects model was used in the meta-analysis, according to which the prevalence of pilocytic astrocytomas was 3.8 (95% CI: 2.3–6.5) (Table 2).

## Discussion

In the present study, the prevalence of primary tumors was investigated worldwide by systematic review and meta-analysis using 78 studies. In his review study, it was found that the highest prevalence of brain tumors is 70.9%. Afterward, neuroepithelial, glioma, meningioma, and glioblastoma tumors have the most significant prevalence, respectively, while finally, medulloblastoma, schwannoma, pilocytic astrocytomas, and oligodendroglial have the least prevalence. The choroid plexus has also the lowest prevalence among primary tumors. In a study conducted by Tamimi et al., In Jordan, the astrocytic glioma tumor was identified as the most common primary tumor, with a prevalence of 37.7% [47]. However, in our study, tumor type was measured as two separate tumors, which ultimately showed that glioma tumors have a prevalence of 31.9% and astrocytic tumors of 21%, which is almost similar to the data obtained from the study done by Tamimi et al. Another study by Johansen et al., it was considered that brain tumors are the most common primary tumors [36] which in our study brain tumors were also measured as the most common primary tumors. In another study by Poschio et al., meningioma and glioblastoma were identified as the most common, with a prevalence of 35% and 26.9%, respectively [29].

Konsel et al. conducted a study to measure the incidence of intracranial tumors in Latin Scotland between 1990 and 1989, which identified 228 primary tumors and 214 secondary tumors. Among the 228 primary tumors, neuroepithelial tumors, which include astrocystic, oligodendroglia, mixed glioma, ependymoma, pineal, and embryonic, were observed in 122 patients (53.5%). Therefore, neuroepithelial tumors were recognized as the most common tumors. Germ cell tumors also showed the lowest prevalence (0.4%). The prevalence study of neuroepithelial tumors is slightly different from our data (34.7%). Our study also presents the lowest prevalence for choroid plexus tumor whose prevalence has not been measured by Consell et al. study [93].

According to studies reported in South Korea, Norway, China, and Jordan, the prevalence of brain tumors was 35, 60.2, 24.56, and 4.4, respectively, which is almost consistent with the results obtained by our study. In most of these studies, brain tumors have a significant prevalence [34–36, 79]. Another primary tumor that had a high prevalence in our study was neuroepithelial tumors which were almost consistent with the previously reported results [25, 29, 53, 54]. It was also observed that the prevalence of choroid plexus tumors is consistent with the present study [47, 67].

It has been observed that the prevalence of primary tumors is increasing in some European countries. Various environmental reasons have been put forward for including, including ionizing radiation, some serum compounds such as N nitrous compounds, air pollution, radio spectrum of electromagnetic waves, and ionizing radiation of the brain, which are among the environmental factors increasing the risk of central nervous system primary tumors. Given the industrial nature of the countries, this seems logical. [94] Among non-European countries, Japan also has a significant prevalence of primary tumors due to radiation from the atomic bomb. In one study, schwannoma was the most common tumor in this country [37], and meningioma was identified as the most common primary tumor in the country [39], showing the important impact of environmental factors on the incidence of central nervous system primary tumors.

Among the cases that measured the prevalence according to age and sex, we can mention the study of Martin Preston, in which it was found that the prevalence of primary tumors is higher in women [65]. A study by Joannstrand et al. found that the prevalence of pituitary tumors in women was higher than in men [43]. Prevalence concerning age has been reported in almost similar studies, and those studies have shown that these tumors are more prevalent in middle-aged people [21, 50, 54, 58].

## Limitation

One of the limitations of this study is the lack of access to data related to age and gender. Also, the lack of access to full text in some studies was another limitation observed in this study.

## Conclusion

As a result, it can be stated that brain tumors are the most common type of primary CNS tumors. It was also observed that tumors involving neuroepithelial cells are more common than other tumors. Since environmental factors are known to be among the factors affecting the prevalence of these tumors, it is necessary to measure the discriminant effect of each of these factors on the prevalence of primary CNS tumors in future studies.

## Data Availability

Datasets are available through the corresponding author upon reasonable request.

## Abbreviations

SID: Scientific Information Database
MESH: Medical Subject Headings
WoS: Web of Science
PRISMA: Preferred Reporting Items for Systematic Reviews and Meta-Analysis
STROBE: Strengthening the reporting of observational studies in epidemiology for cross-sectional study
CNS: Primary central nervous system
